# Social and ecological factors alter stress physiology of Virunga mountain gorillas (*Gorilla beringei beringei*)

**DOI:** 10.1002/ece3.5115

**Published:** 2019-04-01

**Authors:** Winnie Eckardt, Tara S. Stoinski, Stacy Rosenbaum, Rachel Santymire

**Affiliations:** ^1^ Dian Fossey Gorilla Fund Atlanta Georgia; ^2^ Departmet of Anthropology Northwestern University Evanston Illinois; ^3^ Davee Center for Epidemiology and Endocrinology Lincoln Park Zoo Chicago Illinois

**Keywords:** fecal glucocorticoid metabolites, group density, home range, rainfall, temperature

## Abstract

Living in a rapidly changing environment can alter stress physiology at the population level, with negative impacts on health, reproductive rates, and mortality that may ultimately result in species decline. Small, isolated animal populations where genetic diversity is low are at particular risks, such as endangered Virunga mountain gorillas (*Gorilla beringei beringei*). Along with climate change‐associated environmental shifts that are affecting the entire population, subpopulations of the Virunga gorillas have recently experienced extreme changes in their social environment. As the growing population moves closer to the forest's carrying capacity, the gorillas are coping with rising population density, increased frequencies of interactions between social units, and changing habitat use (e.g., more overlapping home ranges and routine ranging at higher elevations). Using noninvasive monitoring of fecal glucocorticoid metabolites (FGM) on 115 habituated Virunga gorillas, we investigated how social and ecological variation are related to baseline FGM levels, to better understand the adaptive capacity of mountain gorillas and monitor potential physiological indicators of population decline risks. Generalized linear mixed models revealed elevated mean monthly baseline FGM levels in months with higher rainfall and higher mean maximum and minimum temperature, suggesting that Virunga gorillas might be sensitive to predicted warming and rainfall trends involving longer, warmer dry seasons and more concentrated and extreme rainfall occurrences. Exclusive use of smaller home range areas was linked to elevated baseline FGM levels, which may reflect reduced feeding efficiency and increased travel efforts to actively avoid neighboring groups. The potential for additive effects of stress‐inducing factors could have short‐ and long‐term impacts on the reproduction, health, and ultimately survival of the Virunga gorilla population. The ongoing effects of environmental changes and population dynamics must be closely monitored and used to develop effective long‐term conservation strategies that can help address these risk factors.

## 1 INTRODUCTION

Rapid environmental changes due to climate change and habitat destruction caused by fast‐growing human populations present major challenges for endangered species conservation. Wild animal populations restricted to small isolated refuges, regardless of their legal protection status, are particularly vulnerable to impacts of persistent anthropogenic disturbances, climate change, and epidemics. Low genetic diversity in “island” populations reduces their ability to adapt to changes, which is exacerbated by their inability to expand or shift their distribution (Belfoire, 2010; McGahey, Williams, Muruthi, & Loubser, 2013). Though species with a wide variety of life history types may be vulnerable, those with slow reproduction rates and long lifespans may particularly struggle to adapt to environmental change, putting them at greater risk of extinction (Belfoire, 2010). While these realities have negative implications both for individual species and for ecosystems, case studies of such situations also provide important opportunities to investigate the ways in which ecological and social change impact larger evolutionary population dynamics.

Physiological parameters, such as stress hormones, are proximate pathways by which evolutionarily relevant changes in health and reproduction can occur. Animals can acclimatize to environmental stressors by adjusting their morphology, physiology, and behavior. For example, many birds and mammals from temperate zones have adapted to high degrees of seasonal variation (Ware et al., 2013; Wingfield & Romero, 2000). Measurable fecal glucocorticoid metabolites (FGM) concentrations in response to such predictable environmental changes, often defined as baseline concentrations (Dickens & Romero, 2013), reflect levels that are required to maintain predictive homeostasis (Romero, Dickens, & Nicole, 2009).

However, living in a rapidly changing environment requires commensurately rapid adaptation and can alter stress physiology on a large scale (Breuner & Hahn, 2003; LaDage, 2015). Nonadaptive stress responses of hypothalamic–pituitary–adrenal (HPA) axis activity (McEwen & Wingfield, 2003) can either decrease FGM concentrations and thus reduced an organism's capacity to react appropriately to a stressor (homeostatic failure), or increase FGM concentrations which can lead to homeostatic overload (Romero et al., 2009). Dramatic changes in FGM concentrations in either direction can negatively affect health, reproductive rates, and mortality. If enough animals in a population are affected, this can cause population decline (Acevedo‐Whitehouse & Duffus, 2009; Boonstra & Singleton, 1993; Dickens & Romero, 2013; Fefferman & Romero, 2013; Friedman & Lawrence, 2002; Leonard, 2007; Romero et al., 2009; Wingfield, 2008).

The pioneering work of Christian and colleagues (Christian, 1950,1956; Christian & Davis, 1964,1966) demonstrated how social environments that affect HPA axis activity in individual mammals can lead to population‐wide consequences. In the decades since that work, evidence of an association between elevated glucocorticoid secretion and social factors, such as high population density and frequent antagonistic interactions (e.g., territorial intrusion and mate competition), has accumulated from laboratory and free‐living animals for a wide range of taxonomic groups, including mammals, birds, reptiles, amphibians, and fish (see Creel, Dantzer, Goymann, & Rubenstein, 2012 for review; Gabriel, Gould, & Cook, 2017). However, social environments can also mitigate individual stress levels. For example, animals with larger numbers of supporters within a social unit during competitive encounters may have lower stress level than peers who have fewer (review by Creel et al., 2012).

In addition to social environment, ecological factors, such as temperature, rainfall, and altitude, can alter HPA axis activity. For example, elevated physiological stress levels have been associated with cold temperatures in chacma baboons (*Papio ursinus*) (Weingrill, Gray, Barrett, & Henzi, 2004), high elevation in white‐crowned sparrow populations (*Zonotrichia leucophrys*), a combination of cold temperatures and high elevation in gelada baboons (*Theropithecus gelada*) (Beehner & McCann, 2008), high temperatures in buffalos (*Bubalus bubalis*) (Megahed, Anwar, Wasfy, & Hammadeh, 2008) and cotton‐top tamarins (*Saguinus Oedipus*) (Ziegler, Scheffler, & Snowdon, 1995), and high temperatures combined with low rainfall in female white‐faced capuchins (*Cebus capucinus*) (Carnegie, Fedigan, & Ziegler, 2011). Among social animals, such ecological factors may interact with social variables to shape hormone profiles (review by Creel et al., 2012). For example, corticosterone levels in subordinate superb starlings (*Lamprotornis superbus*) were higher in years with low rainfall during the pre‐breeding season than in years with high pre‐breeding season rainfall (Rubenstein, 2007). In *Lemur catta*, interpopulation differences in stress levels demonstrated the additive effects of food reduction and high population density (Gabriel et al., 2017).

Using noninvasive methods to monitor individuals’ stress physiology under varying environmental conditions can be a powerful tool to assess overall population health. With adequate longitudinal data, it may be used as an early indicator of imminent population decline (Cockrem, 2005; Hing, Narayan, Thompson, & Godfrey, 2014; Walker, 2005; Wingfield, 2008). Few species conservation programs currently integrate such monitoring, but if it is sustained over time, it can provide important information useful for informing preventative conservation measures to help at‐risk species.

Endangered mountain gorillas (*Gorilla beringei beringei*) (Hickey, Basabose, et al., 2018; Hickey, Granjon, et al., 2018) are vulnerable to extinction for many reasons. Although they are a rare conservation success story whose numbers are increasing in the wild, the world's entire population of slightly over 1,000 individuals lives exclusively in two small isolated fragments of Afromontane forest in the Great Lakes region of Africa. One is in the Virunga massif (455 km^2^), which crosses the borders of Rwanda, Uganda, and Democratic Republic of Congo, and the other is in Uganda's Bwindi Impenetrable National Park (329 km^2^). Both populations are surrounded by high‐density human populations (Bush, Ikirezi, Daconto, Gray, & Fawcett, 2010) that rely primarily on subsistence agriculture. Extreme conservation efforts have led to a recovery of the Virunga population (Robbins et al., 2011), which increased from less than 250 gorillas in the early 1980s to 604 individuals in 2016 (Hickey, Basabose, et al., 2018; Hickey, Granjon, et al., 2018). However, mountain gorilla conservation still faces numerous challenges, due to high levels of anthropogenic pressure on gorilla habitat, disease risk, and ongoing climate change. There are also new challenges emerging as side effects of conservation success, as the gorilla population faces increased intraspecific competition while growing in their confined habitat.

Five decades of monitoring the Virunga population in Rwanda through the Dian Fossey Gorilla Fund's Karisoke Research Center (KRC) has revealed remarkable plasticity in mountain gorilla social structure. While the average gorilla group contains 12.5 individuals (Gray et al., 2013), groups can number up to 65 individuals with up to nine adult males (called silverbacks) at a time (unpublished data from the Dian Fossey Fund International). From the mid‐1990s until 2006, the KRC subpopulation contained three stable multi‐male groups that were well above average size (Caillaud, Ndagijimana, Giarrusso, Vecellio, & Stoinski, 2014). Today, KRC monitors up to eleven groups that originated from those previous three groups, with an increasing proportion of one‐male groups and groups below average size (unpublished data from the Dian Fossey Fund International). Although the Karisoke subpopulation gradually expanded and shifted its range between 2000 and 2011, the growing number of groups has led to a dramatic increase in home range overlap, reduction in home range areas exclusively used by a single group, and to a sixfold increase in annual intergroup encounter rates (Caillaud et al., 2014; unpublished data from the Dian Fossey Fund International). Such encounters can be extremely stressful (Eckardt, Stoinski, Rosenbaum, Umuhoza, & Santymire, 2016) and cause serious injuries or death (Fossey, 1984; Rosenbaum, Vecellio, & Stoinski, 2016; Sicotte, 1993; Watts, 1989).

In addition to substantial changes in their social structure, the Virunga gorilla population, like many faunae, is experiencing the effects of climate change (McGahey et al., 2013). Climate change is predicted to cause higher amounts of less evenly distributed rainfall and warmer temperatures in sub‐Saharan Africa (Advani, 2014). Long‐term climate data obtained from five different sites in Rwanda provide evidence for a significant warming trend in the country since 1977, with a slope up to 0.0445°C per year (Safari, 2012). Although mountain gorillas are tolerant of a wide range of temperatures (Advani, 2014), it is questionable to what extent today's elevation range, which determines their climatological niche, represents the subspecies’ optimum (Thorne et al., 2013). Following extensive deforestation in the 1950s and 1960s at lower elevations around the steep slopes of the six volcanoes forming the Virunga massif, today, the Virunga gorilla population is restricted to extreme elevations of 2,000 to 4,500 m, where temperatures can drop to 0°C. As the population density has grown (Caillaud et al., 2014), an increasing number of groups routinely range at higher elevations (above 3,300 m; Dian Fossey Gorilla Fund unpublished data). This could be a strategy to reduce home range overlap with neighboring groups and the risks associated with intergroup interactions, and/or indicate first behavioral adaptations to warmer temperatures.

These social and ecological changes present a remarkable opportunity to monitor if and how such shifts affect Virunga mountain gorilla physiological parameters, such as baseline stress hormone concentrations. This will create a better understanding of their adaptive capacity to future and current social and environmental variability. We use noninvasive monitoring of FGM, which is widely recognized as an useful measure of stress (Ayres et al., 2012; Millspaugh & Washburn, 2004; Sapolsky, Romero, & Munck, 2000; Walker, 2005) and validated previously in this study population (Eckardt et al., 2016). In addition to establishing the current relationship between social and ecological variation and stress physiology in this gorilla population, these analyses will pave the way for additional longitudinal monitoring for potentially problematic changes in baseline stress levels in this endangered species.

## 2 METHODS

### 2.1 Study site and animals

This study was conducted on the mountain gorillas of the Virunga massif, which is a chain of volcanoes covered with montane cloud forest. The massif's 450 km^2^ spans three countries’ respective parks: Volcanoes National Park (VNP) in Rwanda, the Mikeno sector of Virunga National Park in the Democratic Republic of Congo, and Mgahinga Gorilla National Park in Uganda. All subject animals ranged on the slopes and in the saddle of the Bisoke and Karisimbi volcanoes in VNP, an area that ranges in elevation from 2,300 to 4,500 m. Across elevations, the area contains eight different vegetation types (Grueter et al., 2013). The park boundaries are sharply demarcated by a stone wall, separating the gorillas’ habitat from a dense human population (up to 1,000 people per km^2^; Bush et al., 2010). The climate is typically characterized by mild temperatures year‐round and a bimodal rainfall distribution. Heavy rains last from March to May, with lighter rains from September to November. These alternate with long and short dry seasons lasting from June to August and from December to February, respectively (Mehta & Katee, 2005).

Our data were obtained from 115 gorillas living in 10 habituated social groups which are monitored daily by the Dian Fossey Gorilla Fund International's KRC. All subjects are individually known through nose prints and other physical characteristics. Individuals included both sexes and ranged in age from approximately 1–34 years (mean ± *SD* = 13.2 ± 8.3 years). Social group sizes varied from three to 48 individuals.

### 2.2 Fecal sampling regimen

As part of a long‐term monitoring project initiated in the study population in April 2011 (for detailed methods see Eckardt et al., 2016), we collected weekly fecal samples from each gorilla when the group was not exposed to obvious external stressors (e.g., interunit interactions or being caught in a snare), though some weeks were missed for some individuals due to time limits on data collection. Hereafter, these are referred to as “baseline samples.” Baseline FGM levels maintain reactive homeostasis of the HPA axis through change (Dickens & Romero, 2013; Romero et al., 2009). Using glucocorticoid metabolite measurements from baseline samples is a common approach to studying endocrine response to environmental changes and to identify major stressors in populations (see Creel et al., 2012; Dickens & Romero, 2013 for reviews).

Optimally, such studies should examine changes in stress regulation at multiple levels (Dickens & Romero, 2013), but this is often not practicable in wild settings. For this study, we excluded samples collected after acute stressors for three reasons. First, stressors like intergroup interactions vary strongly in severity, involvement of group members, duration, and outcomes (e.g., female transfers, wounds, distance traveled), which likely causes great variation in individual stress responses that is unrelated to the environmental variation of interest for these analyses (Eckardt et al., 2016). Second, comparing stress response profiles to a specific stressor under varying environmental condition requires complete coverage of fecal sampling six consecutive days post‐event (Eckardt et al., 2016), which is extremely challenging in the wild. Finally, other stressors occur too infrequently (e.g., being caught in a snare, veterinary interventions) to investigate changes in stress response under varying environmental conditions.

### 2.3 Sample collection, hormone metabolite extraction, and enzyme immunoassay

Immediately after defecation, samples were collected in plastic bags, labeled, and transported to the KRC laboratory for storage at −20°C until hormone extraction. FGM extraction occurred in‐country, in most cases approximately 2–3 months after collection. During the study period of 21 months (April 2011–December 2012), we collected a total of 5,525 baseline fecal samples, with a monthly mean (±*SD*) number of baseline samples of 3.03 ± 1.53 per gorilla.

We used a field‐friendly, standardized protocol for fecal hormone extraction that was previously validated for the Virunga gorilla population (Eckardt et al., 2016). Hormone extracts were dried and stored frozen at −20°C until shipping at ambient temperature to the Davee Center for Epidemiology and Endocrinology at the Lincoln Park Zoo (Chicago, IL, USA) for further analysis. FGM concentrations were measured using a biologically validated cortisol enzyme immunoassay (provided by C. J. Munro, University of California Davis, Davis, CA, USA) employing horseradish peroxidase (1:8,500 dilution) ligands and polyclonal antiserum (R4866; 1:20,000 dilution) using established methods (Loeding, Thomas, Bernier, & Santymire, 2011). This cortisol EIA was previously validated for use in Virunga mountain gorillas (Eckardt et al., 2016).

### 2.4 Ecological and social data collection

**Figure 1:**
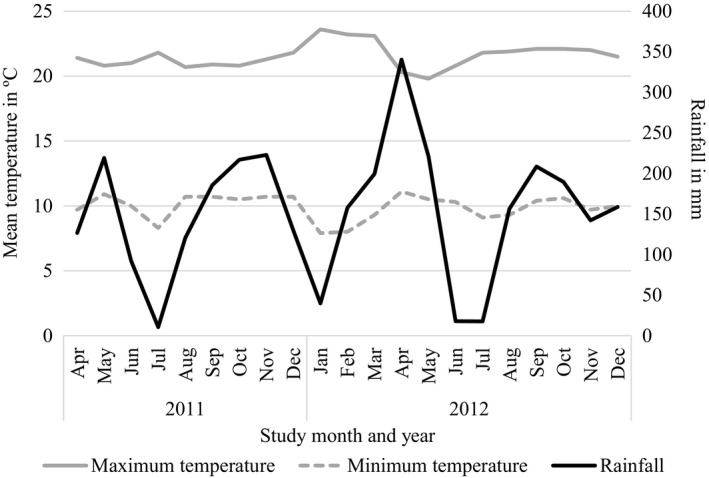
Variation in monthly mean temperature and rainfall from two weather stations near the study area in the Volcanoes National Park across the study period (April 2011–December 2012)

Rainfall data were obtained from the KRC weather station installed at a base camp in Bisate village, adjacent to VNP and close to where most study groups ranged (Figure 1). Information on temperature was provided by the University of Rwanda College of Agriculture, Animal Sciences and Veterinary Medicine, which is based approximately 6 km from the southwestern boundary of VNP, near where study groups are located (Figure 1). Elevation data were collected in each study group four times per day through KRC's long‐term gorilla protection and monitoring program; at the nest site of the previous night, at arrival in the group, at noon, and at the field team's departure from the group. The demographic and behavioral data needed to characterize the gorillas’ social environment were summarized from long‐term records collected as part of KRC's daily monitoring and research.

### 2.5 Home range parameters

Home range size and number of neighboring groups were calculated using KRC long‐term GPS records (*N* = 17,220 GPS points) with Universal Transverse Mercator tracking codes that were collected with handheld GARMIN GPS devices three times daily in each group (at nests where the group spent the previous night, at noon, and at departure of the field team). We applied the fixed kernel density estimation method (see Caillaud et al., 2014) to calculate the 70% kernel home range that was exclusively used by each study group each month. Visual inspections of different kernel home ranges (95%, 90%, 80%, and 70%) indicated that the 70% kernel home range approach best represents habitat usage observed in the field. The sum of all possible pairwise overlap index (OI_1,2_) values (see Caillaud et al., 2014) was calculated to obtain the cumulative home range overlap of each study group with all neighboring groups. Pairwise overlap index is preferable to simple quantitative home range overlap calculations as it incorporates the probability that two groups are actually using the same forest area at a given time (Caillaud et al., 2014). Here we calculated monthly home ranges being exclusively used by each study group as well as monthly sums of pairwise overlap index values and of neighboring groups. All type of kernel home range analyses were conducted using R software version 3.4.1 (R Core Team, 2015) and R package “adehabitatHR” (Calenge, 2015).

### 2.6 Outcome variable, predictors, and control variables

We used baseline samples to calculate mean monthly baseline FGM concentrations for each individual gorilla (minimum samples/month = 3, mean ± *SD* = 4 ± 1). The log values of these monthly means are the outcome variable in our model. The social predictor variables we used included group size, group type (one‐male vs. multi‐male), number of silverbacks present in a group, number of adult females present in a group, number of neighboring groups, home range overlap with neighboring groups, size of home range being exclusively used by a single group, and the number of aggressive interunit interactions (involving social groups or solitary males) a gorilla experienced within a defined period (see statistical analysis below). As discussed above, this analysis does not deal with acute stress responses to specific events, but the number of interunit interactions an animal was exposed to in a given month here serves as proxy for their general level of exposure to the social stimulus of other groups. The number of aggressive interunit interactions a gorilla was exposed to excludes peaceful social interactions between members of two or more social units (e.g., play behavior), as well as interactions where social units could hear or see each but ignored each other's presence (see Mirville et al., 2018).

Ecological factors included in this study were daily minimum and maximum temperature, rainfall, and elevation of the group position. Gorilla sex and age were previously identified as predictors of baseline FGM concentrations in the study population (Eckardt et al., 2016) and were thus also included as control variables.

### 2.7 Statistical analysis

To link log‐transformed monthly mean FGM concentrations of each gorilla with predictors and control variables, we calculated monthly mean values for each numeric predictor (altitude, group size, number of silverbacks in a group, and number of adult females in a group) and rounded them to the nearest whole number, for example mean groups size of 23.7 was rounded to 24. Total rainfall, as well as the number of aggressive interunit interactions, and the number of neighboring groups were summed for each month. All monthly means, monthly sums, monthly home range estimates (exclusively by a group and possible pairwise overlap index), group type (one‐/multi‐male group; coded as dummy variable where 0 = multi‐male group) as well as gorilla sex (coded as a dummy variable where 0 = female), and age were entered as fixed effects in a generalized linear mixed model (Gaussian error distribution, identity link) to examine the relationship between social and ecological factors and monthly mean baseline FGM concentrations of individual gorillas. Because atmospheric temperature reduces with increasing elevation (Körner, 2007), we also tested the interaction effects between elevation and the monthly mean minimum and maximum temperature. The size of the monthly home range exclusively used by a group and monthly pairwise home range overlap index were log‐transformed before model fitting. All continuous predictors and control variables were standardized using z‐transformation (to a mean of zero and a standard deviation of one) to improve the interpretability of model estimates (Schielzeth, 2010). The identity of the individual gorilla and social group were entered as random effects. To avoid inflation of type I and II error rates above the required level of 5% (Schielzeth & Forstmeier, 2009), we included random slope terms where applicable (each combination of fixed and random effects for which fixed effect varies within levels of random effects) to allow for fixed effects and interactions to vary in relationship to each random effect.

Before model fitting, we checked for collinearity between fixed effects by calculating the variance inflation factor (VIF) (Field, 2005) by fitting a standard linear model that excluded the random effects (i.e., individual gorilla and group identification) or interaction terms (i.e., altitude and temperature) using the “vif” function in the R package “car” (Fox et al., 2018). Fixed effects with VIFs higher than the detection criteria of four indicate collinearity issues (https://cran.r-project.org/web/packages/olsrr/vignettes/regression_diagnostics.html). Group size, number of silverbacks, and number of adult females in a group were identified as co‐linear. Therefore, we excluded number of silverbacks and number of adult females in a group as independent variables, but retained group size (summary of final model variables in Table 1).

**Table 1:** Summary of variables entered in the final generalized linear mixed model to examine effects of variation in social and ecological factors on baseline fecal glucocorticoid metabolite concentrations in Virunga mountain gorillas

Model variable	Variable name
Outcome variable	Monthly mean baseline FGM concentration of each gorilla
Random effects	Group identification
	Individual gorilla identification
Fixed effects	
Social predictors	Monthly mean group size (rounded)
	Group type (one‐male vs. multi‐male)
	Monthly home range overlap index with neighboring groups
	Monthly number of neighboring groups
	Monthly home range size exclusively used by a group
	Monthly sum of aggressive interunit interactions
Ecological predictors	Monthly sum of rainfall
	Monthly minimum temperature
	Monthly maximum temperature
	Monthly mean altitude of group location
Control variables	Gorilla sex
	Gorilla age

Model parameters were estimated using maximum likelihood. Model residuals and diagnostics were plotted and checked (see Zuur, Ieno, Walker, Saveliev, & Smith, 2009). Before inspecting the significance of each fixed effect, we tested the significance of the full model by comparing it to a corresponding null model that only included the control variables (i.e., sex and age) as well as both random effects and random slopes (full–null model comparison). This was done by running a likelihood ratio test using the R function ANOVA with the argument test set to “Chisq”. If the full–null model comparison was significant, we derived the significance of each fixed effect with likelihood ratio tests comparing the full model with models reduced by the respective fixed effect using the R function “drop1”. Finally, we checked for model stability by removing random effect levels one at a time from the data set and comparing the estimates of each fixed effect obtained from these data subsets to those estimates obtained from the full data set; estimates of fixed effects remained constant across observations without any stability issues being detected.

## 3 RESULTS

As previously shown (Eckardt et al., 2016), females had significantly higher baseline FGM concentrations than males, and older animals had lower baseline FGM levels than younger animals (Table 2).

**Table 2:** GLMM output: influence of social and ecological factors on monthly mean baseline fecal glucocorticoid metabolite concentrations in Virunga mountain gorilla individuals (*N* = 1,094 gorilla‐month). significance level *p* < 0.05

Fixed effects	Estimate	*SE*	CI lower	CI upper	*t*	*p*‐value
Sex: males	−0.057	0.024	−0.101	−0.012	−2.41	**0.027**
Age	−0.036	0.012	−0.067	−0.010	−3.01	**0.009**
Rain	0.058	0.022	0.011	0.102	2.64	**0.020**
Temperature min	0.045	0.016	0.012	0.076	2.80	**0.017**
Temperature max	0.055	0.023	0.010	0.102	2.39	**0.031**
Altitude	0.042	0.050	−0.058	0.143	0.85	0.402
Exclusive home range	−0.048	0.018	−0.085	−0.010	−2.67	**0.046**
Number of interactions	−0.011	0.011	−0.033	0.012	−1.02	0.340
Number of neighboring groups	0.034	0.027	−0.026	0.088	1.24	0.232
Home range overlap	0.016	0.039	−0.095	0.064	0.40	0.232
Group size	0.047	0.047	−0.048	0.148	1.00	0.327
Group type: one‐male	0.100	0.082	−0.059	0.275	1.23	0.232

### 3.1 Relationship between ecological factors and baseline FGM levels

**Figure 2:**
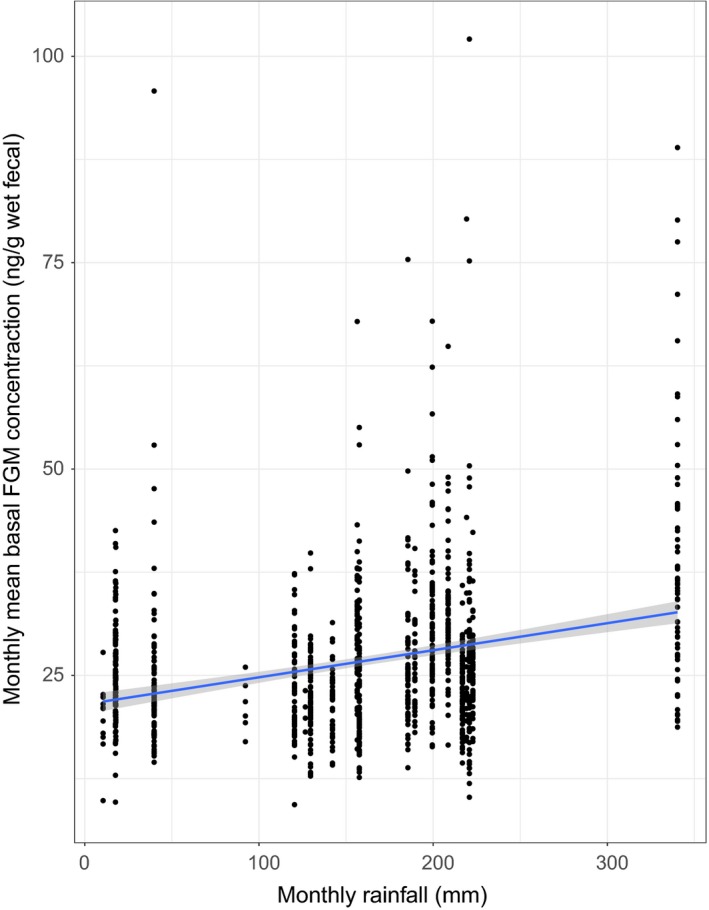
Relationship between monthly rainfall and monthly mean baseline fecal glucocorticoid metabolite (FGM) levels

Mean baseline FGM levels were significantly higher in months that had higher rainfall than in months that had lower rainfall (Table 2; Figure 2). Additionally, baseline FGM levels were significantly higher when the monthly mean maximum and minimum temperature was higher. Neither the interaction terms involving mean elevation nor the main effect of mean elevation were related to baseline FGM levels.

### 3.2 Relationship between social environment and baseline FGM levels

**Figure 3:**
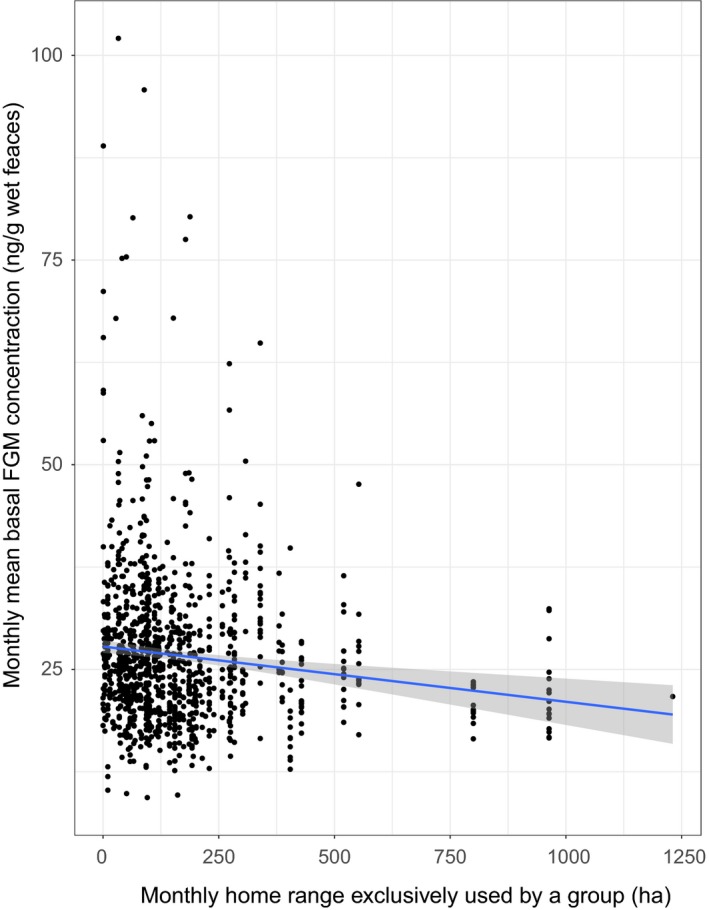
Relationship between monthly home range size exclusively used by a group and monthly mean baseline fecal glucocorticoid metabolite levels

Of all test variables representing the social environment, only the size of the monthly homes range that was exclusively used by a study group explained monthly mean levels of baseline FGMs. Larger exclusive areas predicted lower baseline FGM levels (Figure 3). There was no evidence that variation in monthly numbers of aggressive interunit interactions, number of neighboring groups, monthly pairwise overlap indices with neighboring groups’ home range, or mean group size, or group type affected the gorillas’ baseline FGM levels.

## 4 DISCUSSION

We found evidence that baseline FGM levels in the Virunga mountain gorilla subpopulation were related to both social and ecological variables, but that ecological variation was generally a better predictor of baseline FGM levels than is social variation. Overall, we found little relationship between the wide variation in the gorillas’ social environment and their physiological stress levels. Since different social configurations are associated with different costs and benefits (Robbins & Robbins, 2005; Robbins, Stoinski, Fawcett, & Robbins, 2009; Robbins, Robbins, Gerald‐Steklis, & Steklis, 2007; Watts, 2000), it could be that no one type is clearly connected to lower (or higher) physiological stress. It is also possible that detectable alteration of baseline FGM levels in the study subpopulation during the changes observed in group structures and group densities since 2006 may have already occurred before this study was implemented in 2011. In contrast, both temperatures and rainfall predicted baseline FGM values, though elevation did not.

Previous research found that for the Bwindi mountain gorilla population, mutually exclusive home range areas are characterized by high food availability, and that daily movement decisions are made based on avoiding interactions with neighboring groups (Seiler et al., 2017). Extrapolating to the current results, higher baseline FGM levels found in Virunga gorillas ranging in groups with a small or no mutually exclusive home range area might result from reduced feeding efficiency and increased travel efforts to actively avoid neighboring groups. These two factors could lead to a higher risk of imbalance between energy intake/reserves and energy requirements, causing a rise in allostatic load (McEwen & Wingfield, 2003; Romero, 2004). However, a comparative study on spatial and temporal gorilla group ranging patterns in the Virunga population is needed to better understand whether the dynamics at work in Bwindi cause the relationship between elevated baseline FGM levels and reduced exclusive home range areas in the Virungas. There are possible alternative explanations. For example, the size of the home range area exclusively used by a group may also reflect the level of risk of exposure to threats from neighboring groups. Data from other species suggests such conditions can cause individuals to mount a stress response even in absence of the actual stimuli (LaDage, 2015; Romero, 2004), such as interunit interactions. Apart from visual interunit interactions, chest‐beats, screams, and visual signs of groups passing through an area may inform gorillas about their social environment (Seiler et al., 2017).

Interunit interactions can cause acute elevation of FGM levels in mountain gorillas (Eckardt et al., 2016). Such interactions have increased sixfold in the study subpopulation of Virunga mountain gorillas between 2006 and 2011 (Caillaud et al., 2014). Our findings suggest that baseline FGM levels were not affected even after exposure to up to five aggressive interactions per month. This is not unprecedented in primates; for example, there was also no correlation between stress levels and intergroup interaction rates in wild black‐chested mustached tamarin (*Saguinus mystax*) in Perú (Huck, Löttker, Heymann, & Heistermann, 2005). However, interunit interactions will likely continue to rise as the Virunga population grows and may create a permanent environment of increased intraspecific threats. Therefore, stress physiology needs to be closely monitored in the Virunga population as their interunit interaction rates and group densities continue to climb. We recommend expanding stress hormone monitoring to areas in the forest where group densities are still relatively low compared to where the study subpopulation ranges (Gray et al., 2010,2013). If current trends continue, these are likely to reach similar densities and undergo similar changes in the population social structure.

In addition to variation in social environment, variation in climatic factors was associated with change in baseline FGM levels. Individuals who experienced more rainfall and higher daily minimum and maximum temperatures had higher baseline FGM levels. There could be several explanations for this result. During heavy rain, mountain gorillas sit still in a huddle, but if rain persists, they will resume feeding and compensate for lost feeding time (Watts, 1988). Thus, reduced energy intake on rainy days is unlikely to explain elevated baseline FGM levels, as has been shown in food‐deprived humans (Tomiyama et al., 2010) and birds (Kitaysky, 2001; Kitaysky, Piatt, Wingfield, & Romano, 1999). However, huddling during rain may also function as thermoregulatory behavior (Watts, 1988), similar to Japanese macaques (*Macaca fuscata*) using hot spring bathing to counter cold stress during the winter (Takeshita, Bercovitch, Kinoshita, & Huffman, 2018). Resumption of feeding during heavy or prolonged rain might therefore increase allostatic load, as the gorillas work harder to maintain a stable body temperature (McEwen & Wingfield, 2003; Romero, 2004).

Higher monthly mean maximum and minimum temperatures were also associated with higher baseline FGM levels. As for rainfall, similar physiological and behavioral processes may act to keep body temperature stable. On hot and sunny days, mountain gorillas often seek shade in the understory vegetation. This may also translate into longer resting periods and reduced time spent feeding, as has been shown in vervet monkeys (*Chlorocebus pygerythrus*) during periods of high temperature (McFarland, Barrett, Boner, Freeman, & Henzi, 2014). VNP is located near the equator, where sunlight strikes the earth's surface at a perpendicular angle. This causes highly concentrated solar radiation, which is further magnified at high elevations (Clegg & Harrison, 1970). Thus, mountain gorillas may experience heat stress and reduced energy intake during long dry and sunny periods. Their black hair may increase the risk of experiencing thermal stress, as has been shown in equator‐dwelling goats with dark coat colors (Acharya, Gupta, Sehgal, & Singh, 1995).

Unlike in another high‐elevation dwelling primate, Gelada baboons (*Theropithecus gelada*) (Beehner & McCann, 2008), and in female chacma baboons (*Papio hamadryas ursinus*) (Weingrill et al., 2004), cold temperatures and elevation were unrelated to baseline FGM concentrations in mountain gorillas. This suggests mountain gorillas are better adapted to cold temperatures than to warm ones. Mountain gorillas use various behavioral strategies that appear to mitigate cold stress. At night when temperatures drop, they huddle in their nests. The vegetation they use to build nests (Schaller, 1963) provides insulation from moist ground (Tutin, Parnell, White, & Fernandez, 1995), and the surrounding vegetation and slopes shelter them from rain and wind. After very cold and rainy nights, gorillas stay in their night nests longer than usual in the morning, presumably to prevent cold stress. However, in 2010 an adult female in a group that was ranging at 3,300 m died from suspected hypothermia on a cold, rainy night (DFGFI/Gorilla Doctors unpublished data). The use of monthly mean values in our study may mask physiological responses to single days with extreme temperatures. Future studies are needed to better understand immediate stress responses to extreme weather occurrences, such as exceptionally cold nights and days, or periods of extreme heat or heavy rainfall. The lack of a relationship between elevation and FGM also hints that variation in food biomass across different habitat types along the elevation gradient (Watts, 1998) has no negative impacts on energy intake, which is consistent with a study on energy balance in mountain gorilla females (Grueter, Deschner, Behringer, Fawcett, & Robbins, 2014). Mountain gorilla diet is highly flexible which uniquely equips them to use a wide range of elevation and adapt to varying food availability (Ganas, Robbins, Nkurunungi, Kaplin, & McNeilage, 2004; McNeilage, 2001).

Overall, our findings on the relationship between ecological factors and stress response suggest that Virunga mountain gorillas may have a harder time coping with warmer temperatures and more extreme rainfall. Thus, mountain gorillas might be more sensitive to warming trends than previous research has suggested (Thorne et al., 2013), since their small habitat restricts their ability to seek out colder temperatures (Loarie et al., 2009; La Sorte & Jetz, 2010). Climate models predict a temperature increase of 3.6°C, prolonged dry seasons, and a potential upward shift of flora communities of approximately 600 to 700 m in the region by 2090, relative to 1990 (McGahey et al., 2013; Warner et al., 2015). Additionally, rainfall trends in Rwanda indicate less evenly distributed rain, with overall shorter wet seasons and more extreme swings between the wet and dry seasons, especially in the Northern Province where the Virunga massif is located (Warner et al., 2015). Longer, warmer dry seasons combined with more concentrated rainfall also lead to decreased net primary plant productivity (Thorne et al., 2013) and thus may negatively affect food availability and regeneration. Changes in the availability, biomass, and phenology of key gorilla food plant species need to be closely monitored along with gorilla population trends, ranging patterns, and stress response (Grueter et al., 2013).

Each single identified social and ecological factor associated with elevated baseline FGM levels in Virunga mountain gorillas may not meaningfully impact reproduction and long‐term survival of this wild ape population. However, multiple factors acting simultaneously may profoundly change the amount of physiological stress experienced by the average individual, which could have both short‐term and long‐term detrimental effects on population size (Acevedo‐Whitehouse & Duffus, 2009; Fefferman & Romero, 2013; Marasco et al., 2018). Additive costs resulting from interacting factors could further aggravate outcomes at the population level.

For example, climate change coupled with continuing gorilla population growth could negatively affect the regeneration of gorilla food plants and nutrient availability, elevating baseline gorilla FGM levels and subsequently compromising immunocompetence (Acevedo‐Whitehouse & Duffus, 2009). Impaired immunocompetence increases disease susceptibility and thus may have a detrimental effect on the viability of the mountain gorilla population (see Friedman & Lawrence, 2002; Leonard, 2007). This is particularly worrisome in light of newly emerging and faster‐spreading diseases that may be one result of climate change in the region (Belfoire, 2010). Those combined with higher gorilla densities (Hickey, Basabose, et al., 2018; Hickey, Granjon, et al., 2018), which better facilitate disease transmission, create a potentially problematic environment.

As with all critically endangered fauna, low heterozygosity in this species (Xue et al., 2015) leaves the Virunga gorilla population with few genetic resources to adapt to environmental change (see Bijlsma & Loeschcke, 2012; Schierenbeck, 2017). Therefore, it is of utmost importance to closely monitor the ongoing effects of environmental changes and population dynamics. Mountain gorilla survival will depend on our ability to adapt conservation strategies to quickly changing environments. In the short term, conservationists need to identify and alleviate existing stressors and threats that are unrelated to climate change and natural gorilla population growth, which will indirectly help mitigate negative effects of a rapidly changing environment on mountain gorillas’ long‐term survival (Advani, 2014).

## AUTHORS’ CONTRIBUTION

All authors contributed to the study design. SR and WE orchestrated and contributed to data collection. SR and RS orchestrated the hormone analysis. We executed all analysis advised by SR, RS, and TS. We developed the first manuscript draft, which was subsequently edited and approved by all authors.

## Data Availability

Data entered into the model of this study are archived in Dryad Data Repository (https://doi.org/10.5061/dryad.0md816p).
